# In Silico Study towards Repositioning of FDA-Approved Drug Candidates for Anticoronaviral Therapy: Molecular Docking, Molecular Dynamics and Binding Free Energy Calculations

**DOI:** 10.3390/molecules27185988

**Published:** 2022-09-14

**Authors:** Wesam S. Qayed, Rafaela S. Ferreira, José Rogério A. Silva

**Affiliations:** 1 Medicinal Chemistry Department, Faculty of Pharmacy, Assiut University, Assiut 71526, Egypt; 2 Laboratório de Planejamento e Desenvolvimento de Fármacos, Instituto de Ciências Exatas e Naturais, Universidade Federal do Pará, Belém 66075-110, Brazil

**Keywords:** anti-COVID-19, drug repositioning, molecular docking, molecular dynamic simulations, binding free energy

## Abstract

The SARS-CoV-2 targets were evaluated for a set of FDA-approved drugs using a combination of drug repositioning and rigorous computational modeling methodologies such as molecular docking and molecular dynamics (MD) simulations followed by binding free energy calculations. Six FDA-approved drugs including, Ouabain, Digitoxin, Digoxin, Proscillaridin, Salinomycin and Niclosamide with promising anti-SARS-CoV-2 activity were screened in silico against four SARS-CoV-2 proteins—papain-like protease (PLpro), RNA-dependent RNA polymerase (RdRp), SARS-CoV-2 main protease (Mpro), and adaptor-associated kinase 1 (AAK1)—in an attempt to define their promising targets. The applied computational techniques suggest that all the tested drugs exhibited excellent binding patterns with higher scores and stable complexes compared to the native protein cocrystallized inhibitors. Ouabain was suggested to act as a dual inhibitor for both PLpro and Mpro enzymes, while Digitoxin bonded perfectly to RdRp. In addition, Salinomycin targeted PLpro. Particularly, Niclosamide was found to target AAK1 with greater affinity compared to the reference drug. Our study provides comprehensive molecular-level insights for identifying or designing novel anti-COVID-19 drugs.

## 1. Introduction

Severe acute respiratory syndrome coronavirus 2 (SARS-CoV-2) caused the well-known coronavirus disease of 2019, COVID-19 [1]. The World Health Organization (WHO) has declared a public health emergency of worldwide importance [2]. The fundamental difficulty is that COVID-19 is a very contagious disease with a high fatality rate [3]. It has exhibited strong transmissibility and has caused a substantial number of deaths worldwide since its inception. It is still creating havoc all around the world [4]. This signifies that appropriate treatment is required as soon as an epidemic begins in order to prevent the disease from spreading [2,5].

The global research and clinical communities have been under tremendous pressure to produce treatments to battle the ever-growing pandemic since the outbreak of the unique COVID-19 disease. However, drug development can take several years and there is no certainty that it will be completed successfully. Furthermore, with the existing drug production or application method, this cannot be done, and it takes several years for newly approved drugs to reach the market. Instead, SARS-CoV-2 infection could be treated with existing drugs. When it comes to developing new drugs/vaccines or repurposing approved ones, detailed information on the possible target(s) is essential [6]. 

Since the start of the pandemic, scientists have been working on developing vaccinations against SARS-CoV-2. As of July 2021, the WHO had approved 14 vaccinations under the Emergency Use Listing (EUL), with many more on the waiting list. Although all the licensed vaccinations have proven to be rather effective up to this point, the emergence of new strains due to rapid virus mutations poses a significant imminent danger to vaccine efficacy. Various routes are being targeted to limit viral replication inside human cells, and studies are being conducted to understand the complexities of how the virus affects the human body [7].

Potential anti-coronavirus drugs can be divided into two categories based on the target: One targets the human immune system or human cells, while the other targets the coronavirus itself. The innate immune system response is critical in suppressing coronavirus replication and infection in the human immune system, and interferon is believed to improve the immune response [8]. Furthermore, inhibiting viral endocytosis is pivotal in COVID-19 therapy [9]. The therapies that target the coronavirus include blocking the virus’s binding to human cell receptors or inhibiting the virus self-assembly process by acting on some structural proteins, as well as preventing the synthesis of viral RNA by acting on the virus genetic material or inhibiting virus replication by acting on critical viral enzymes [10]. The spike protein, membrane protein, envelop protein, nucleocapsid protein, protease, helicase, and hemagglutinin esterase are the seven key structural protein targets for SARS-CoV-2 [11], while replicase proteins, polyprotein 1a (PP1a) and polyprotein 1ab (PP1ab) represent the other 16 nonstructural proteins (NSPs) formed from the virus [12]. All NSPs have a unique role in replication and transcription. On the other hand, human targets, including many kinases, e.g., adaptor-associated kinase 1 (AAK1), cathepsin L, and the transmembrane serine protease 2 (TMPRSS2), are involved in the advancement of symptoms associated with SARS-CoV-2 infections, such as pneumonia, inflammation, and fibrosis [13]. Kinases react to and control the synthesis of potentially damaging cytokines (IL-6, IL-10, and TNF) and proteins linked to inflammation and the induction of pulmonary fibrosis (such as the pro-inflammatory cytokine TGF-). Thus, kinase inhibitors can have antiviral, anti-inflammatory, cytokine-suppressive, and antifibrotic properties, all of which could be beneficial in the fight against respiratory viral infections. Several kinase inhibitors are now being investigated in COVID-19 clinical studies [13,14]. 

Because there is a pressing need to treat the disease, most research is focusing on repurposing already approved drugs rather than classical drug discovery. Remdesivir was the first of all the COVID-19 drugs to be approved for hospitalized patients by the US Food and Drug Administration (FDA). The concept of drug repositioning, also known as drug repurposing, drug reprofiling, and therapeutic switching, has gained considerable attention from pharmaceutical companies and researchers due to its potential to discover new uses for approved drugs [15]. Compared to traditional de novo methods, the approach is more efficient in terms of time and cost savings. The shorter development time, in particular, corresponds to an example of the general scenario, in this case, COVID-19. Furthermore, contemporary and powerful artificial intelligence (AI) technologies have substantially improved the throughput and accuracy of drug repurposing [16]. A multi-target molecular modeling protocol was employed to identify druggable targets associated with viral replication, in addition to a molecular dynamic simulation against the SARS-CoV-2 viral genome, as mutations are one of the most challenging obstacles to overcome with antiviral therapeutics [17,18].

Since the outbreak started, a flood of computational articles identifying potential antiviral drug repurposing candidates has appeared in peer-reviewed journals and preprint services, but most of them lack experimental confirmation [10,19,20,21,22,23]. The purpose of this research was to look at the primary SARS-CoV-2 proteins as potential targets for active FDA-approved drugs with a more enhanced antiviral profile with the aid of computational tools, docking, and molecular dynamic simulations. As the selected listed drugs have been previously experimentally screened against the SARS-CoV-2 virus, we tried to elucidate their activity to find their viral targets from structural and nonstructural protein targets, which are crucial for viral replication and translation, as well as one of the promising human kinases. These drugs can be used in future research as a potential pharmacophore scaffold for the development of promising anti-COVID-19 therapies.

## 2. Results and Discussion

### 2.1. Rationale of Drug and Proteins Selection 

A public health emergency with regard to COVID-19 in all countries and efforts to develop an effective vaccine or drug for prevention or treatment are critical. New compound approval as a drug necessitates a lengthy investigation period and a considerable financial investment. So, the best starting point is repurposing already approved drugs when developing novel COVID-19 therapeutics. We virtually reprofiled drugs against multiple SARS-CoV-2 targets, including SARS-CoV-2 Mpro, PLpro, and RdRp, in addition to AAK1 as a human target.

Previously, Meehyun Ko et al. tested a total of 24 FDA-approved drugs against SARS-CoV-2 in Vero cells and Calu3 cells—a well-known human lung cell line—as potential antiviral candidates [24,25]. For deep computation, we created a panel of the most active (lC_50_ less than 2.5 μM) FDA-approved drugs that showed antiviral activity compared to the clinically applied drugs Chloroquine, Remdesivir, and Lopinavir (IC_50_ in Vero cells 11.4, 7.28 and 9.12 μM, IC_50_ Calu cells, respectively). The current study tried to find the potential target for each drug against SARS-CoV-2 through virtual screening. The included drugs are currently on the market and some are in clinical trials against SARS-CoV-2 infections. The drugs that are currently in clinical studies were given special consideration. The drugs’ generic name, clinical use, and reported activity against SARS-CoV-2 (IC_50_ μM) are presented in Table 1.

### 2.2. Molecular Docking

The structures of four SARS-CoV-2 proteins (macromolecules) and antiviral drugs (ligands) were retrieved and optimized for the docking simulation to investigate the binding preference between the protein binding pocket residues and ligands. The molecular docking resulted in ten different docked poses for each drug. Each drug’s optimal pose was chosen based on how closely its binding pattern in the active pocket matched that of the original ligand, taking into consideration the binding energy. Accordingly, the drugs under investigation were scored, with the top scorers being those with the lowest binding energy. For each ligand, the complex with the best-predicted affinity energy (AE) was used for the MD simulations.

**Scheme 1:**
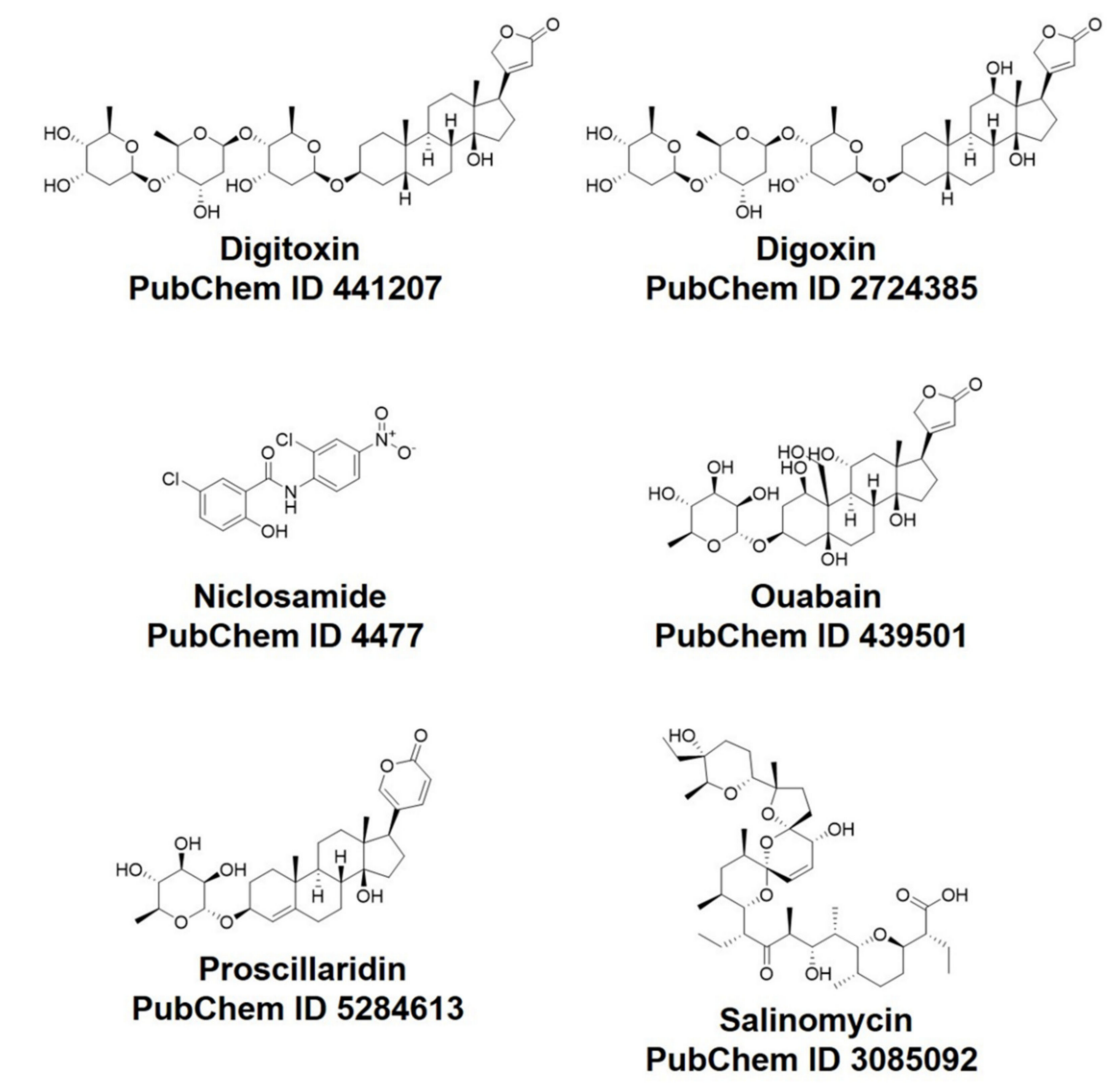
FDA-approved drugs that were included in the virtual study.

In this research, the docking efficacy of six FDA-approved drugs previously tested against SARS-CoV-2 (Scheme 1) against a host and viral SARS-CoV-2 proteins was studied. Four SARS-CoV-2 proteins were investigated. Among them, three are viral SARS-CoV-2 proteins: Mpro, PLpro, and RdRp. In addition to human protein AAK1, docking studies were conducted using MOE software. The MOE software’s output was further evaluated and displayed. These docking studies predicted the AE for the compounds against the studied SARS-CoV-2 systems listed in Table 2.

The repositioned drug with the highest score for each investigated target was selected. Ouabain (OUB) showed the greatest affinity for SARS-CoV-2 Mpro and PLpro (−8.21 and −9.52 Kcal/mol, respectively) contrasted to the enzyme native ligand X77 and GRM (−8.05 and −8.46 Kcal/mol), respectively. DGX and DIG showed AEs comparable to RdRp with binding energy (−7.63 and −7.69 Kcal/mol, respectively) compared to cocrystallized ligand Remdesivir (RDV) which revealed AE equal to −7.8 Kcal/mol. Salinomycin (SLM) showed binding affinity for PLpro (AE = −9.05 Kcal/mol) compared to cocrystallized ligand GRM (AE = −8.46 Kcal/mol). Niclosamide (NIS) showed excellent interaction and binding affinity (AE = −11.84 Kcal/mol) for the AAK1 system in comparison to its cocrystallized ligand LKB (AE = −11.72 Kcal/mol). Finally, Proscillaridin (PRO) showed optimum binding affinity for RdRp (AE = −7.11 Kcal/mol).

### 2.3. Analysis of the Intermolecular Binding Pattern of Repurposed Systems by Docking and MD Simulations

Molecular docking is a powerful approach for evaluating binding affinity and investigating the binding pattern of ligands that bind to the active region of target proteins [26]. The most likely interaction of the ligand with the protein receptor was identified and visualized using molecular docking studies performed using MOE software. Both the binding modalities and affinity of docked ligands at the active pocket of the protein were predicted using the docking score and hydrogen bonds generated between amino acids and the interacted atoms. A specific ligand binds and interacts at the active site residues of a target protein with a certain affinity and strength that is referred to as the binding energy. A docking investigation was conducted for FDA-approved drugs: OUB, DGX, DIG, SLM, NIS, and PRO using MOE software, as discussed above.

Although molecular docking may shed light on repurposing drug protocols [27], their usual fast and approximate algorithms lack protein flexibility, which may be related to the recognition and binding involved in ligand–protein complexes [28]. In this sense, molecular dynamic (MD) simulation techniques, which are more computationally expensive but more accurate than docking protocols, may provide a better complementary result [29]. In summary, MD simulations can be used to study the macromolecule characteristics and provide a suitable ensemble for thermodynamics analysis.

Here, MD simulation calculations were conducted to understand the structural stability of some favorable docking results. We can summarize it as (i) PLpro–GRM, PLpro–OUB, and PLpro–SLM systems; (ii) Mpro–X77 and Mpro–OUB systems; (iii) RdRp–RDV and RdRp–DIG systems; (iv) AAK1–LKB and AAK1–NIS systems. Initially, The RMSD analysis from the 200 ns of MD trajectories were explored to examine flexibility across the simulation periods.

#### 2.3.1. PLpro Systems 

**Figure 1:**
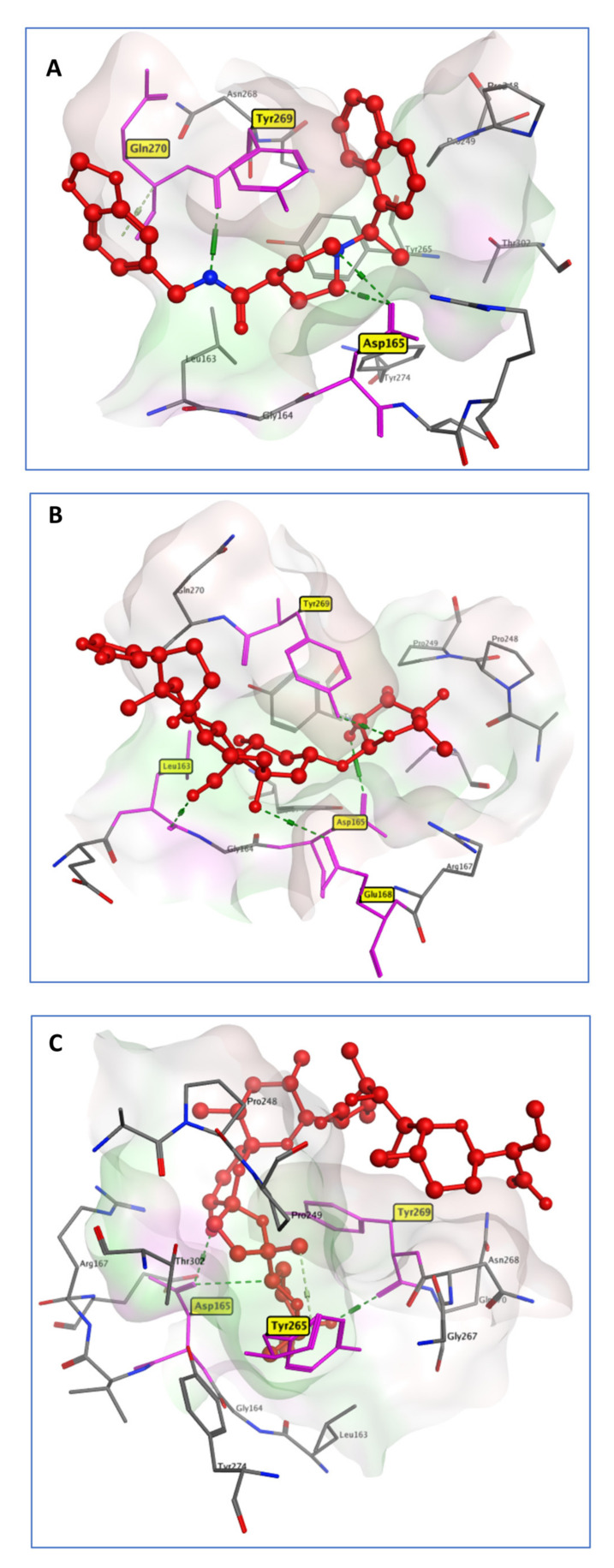
3D model of the interactions between cocrystallized ligand GRM (**A**), OUB (**B**), and SLM (**C**) with PLpro active site residues.

PLpro is an essential aspect of the replicase–transcriptase complex as a nonstructural protein 3 (NSP3). By releasing NSP1–3 from the viral polyprotein, which is required for viral replication, human proteases with a comparable cleavage selectivity can be known, and inhibitors of that enzyme are unlikely to be harmful [30]. The comprehensive examination of the Mpro catalytic mechanism makes it a promising target for anti-COVID-19 medication development [30]. PLpro acts as a negative regulator of the immune response to viral infection [31]. All of these factors combine to make it an attractive target for antiviral drugs. PLpro was represented with the cocrystallized lead compound GRM [32]. Of the six selected drugs on the active pocket of PLpro, Ouabain (OUB) was approved by the FDA to treat heart conditions of patients for over 10 decades. OUB showed the highest docking score (−9.52 Kcal/mol) among the tested compounds, which was higher than that of the native ligand GRM (-8.46 Kcal/mol). GRM hydrogen-bonded with the carboxylate of Asp165 and with the backbone carbonyl oxygen of Tyr269. OUB rings similarly showed binding to the PLpro active pocket Asp165 and Tyr269. In addition, it showed an extra hydrogen-bond interaction with Leu163 and Glu168 (Figure 1). This outcome is consistent with in vitro screening as it has the lowest IC_50_ values of all the tested drugs.

*Streptomyces albus* produces Salinomycin (SLM), a carboxylic polyether ionophore. Ionophores have a wide range of bioactivity, including antibacterial, antifungal, antiparasitic, antiviral, and, more recently, anti-tumor properties [33]. In a trial to reposition SLM for the treatment of COVID-19, it showed antiviral activity, with a lower IC_50_ compared to the control drugs. This activity may be attributed to the SLM binding affinity for PLpro, as manifested from the docking simulation. SLM is connected to key PLpro residues through three hydrogen bonds (Figure 1). It bonded to the Asp165, Tyr265, and Tyr269 amino acids with a docking score of −9.02 Kcal/mol, which is more than that of the crystallized ligand (8.46 Kcal/mol).

**Figure 2:**
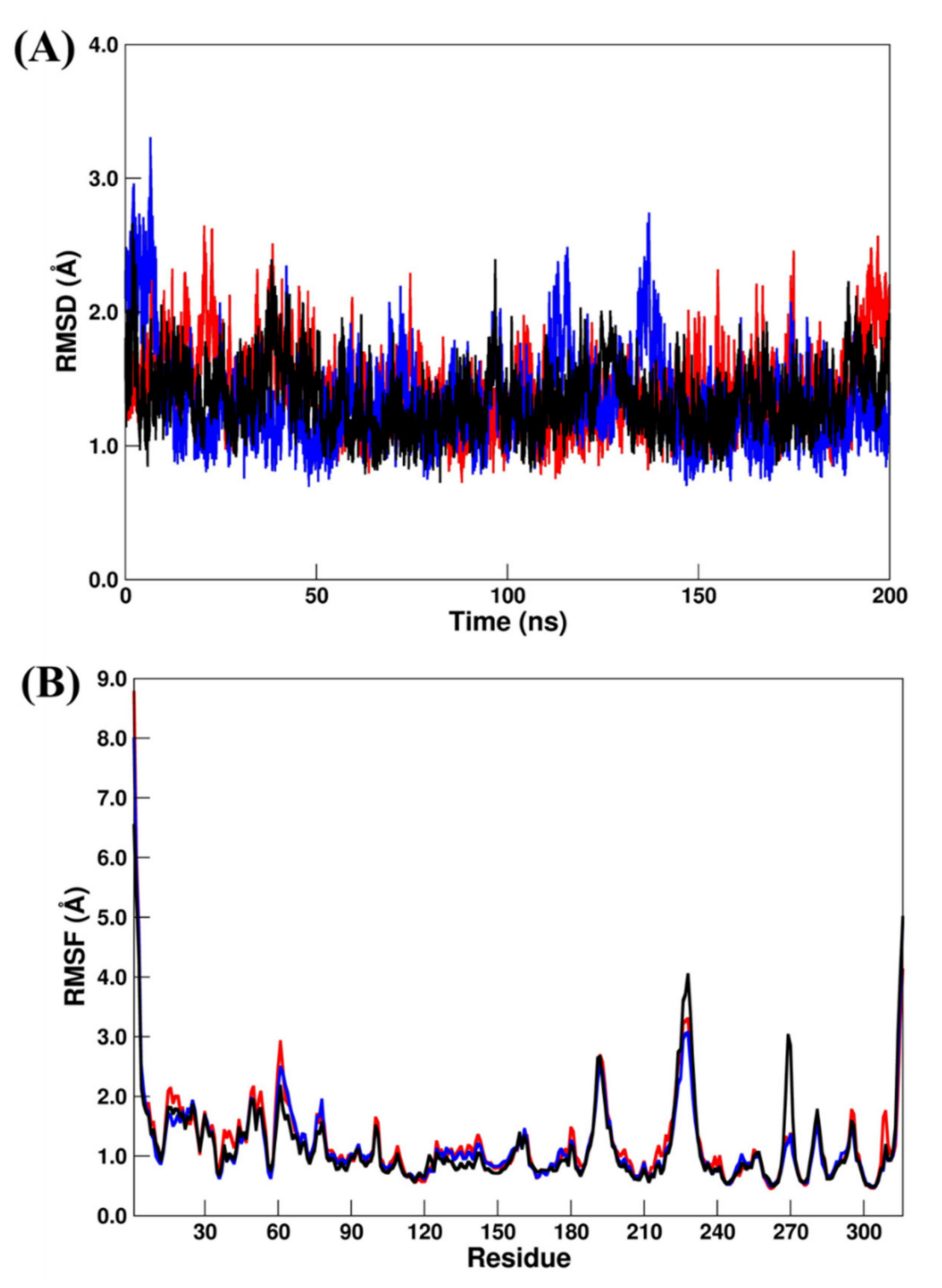
(**A**) RMSD and (**B**) RMSF plots for PLpro–GRM (red line), PLpro–OUB (blue line), and PLpro–SLM (black line). All values are reported in Å.

A suitable structural stabilization for PLpro systems by computing RMSD/RMSF can be observed in Figure 2A,B, where all systems converged to their respective average structures during the total MD simulation scale. In addition, all the systems showed very small RMSDs (Figure 2A), which changed from 1.41 ± 0.30 Å (PLpro–GRM) to 1.33 ± 0.34 Å (PLpro–OUB) and 1.34 ± 0.24 Å (PLpro–SLM), respectively. From the RMSF analysis (Figure 2B), the values changed from 1.24±0.80 Å (PLpro–GRM) to 1.17 ± 0.74 Å (PLpro–OUB) and 1.18 ± 0.70 Å (PLpro–SLM), respectively. All these results suggest that the crystal (PLpro–GRM) and repurposed (PLpro–OUB and PLpro–SLM) systems were in a very similar conformation during the MD simulation.

#### 2.3.2. Mpro Systems 

**Figure 3:**
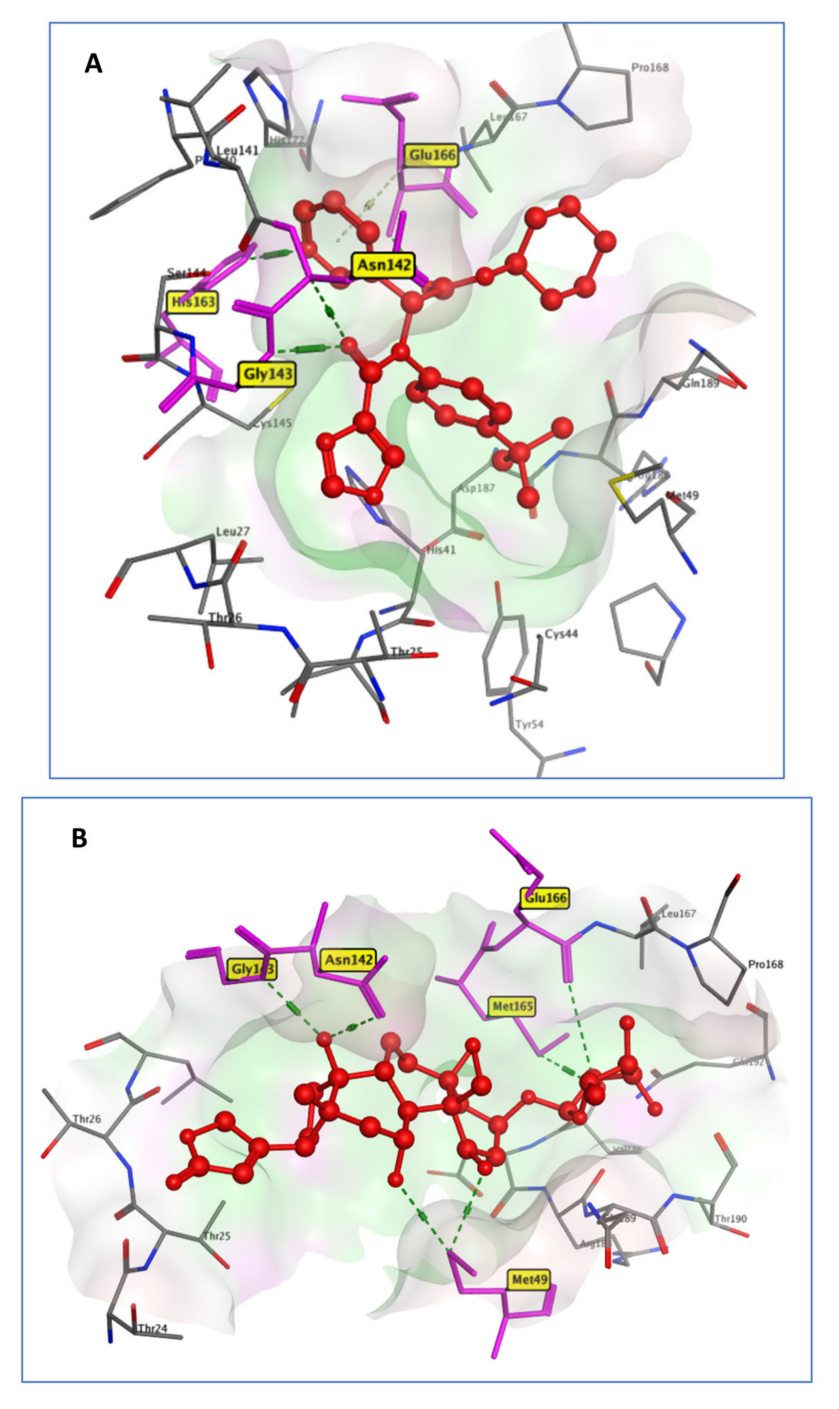
3D model of the interactions of cocrystallized ligand X77 (**A**) and OUB (**B**) with Mpro active site residues.

The comprehensive examination of the Mpro catalytic mechanism identified it as a promising target for anti-COVID-19 medication development. For Mpro systems, it should be highlighted that previous experimental studies show that in the biological environment, Mpro acts as a dimer instead of a monomer [34]. However, for computational analysis and computer-aided drug design strategies, only the monomeric form is necessary [35,36,37]. For the comparison of the docking data of the studied compounds, the cocrystallized ligand X77 (N-(4-tert-butylphenyl)-N-[(1R)−2-(cyclohexylamino)−2-oxo−1-(pyridine−3-yl)ethyl]−1H-imidaz-ole−4-carboxamide) was employed as a reference ligand. It interacted with Asn142, Gly143, His163, and Glu166, through four hydrogen bonds [38], while OUB created six hydrogen bonds with Met49, Asn142, Gly143, Met165, and Glu166 (Figure 3). It exhibited a binding score of −8.21 Kcal/mol, similar to that of the reference ligand (−8.05 Kcal/mol).

**Figure 4:**
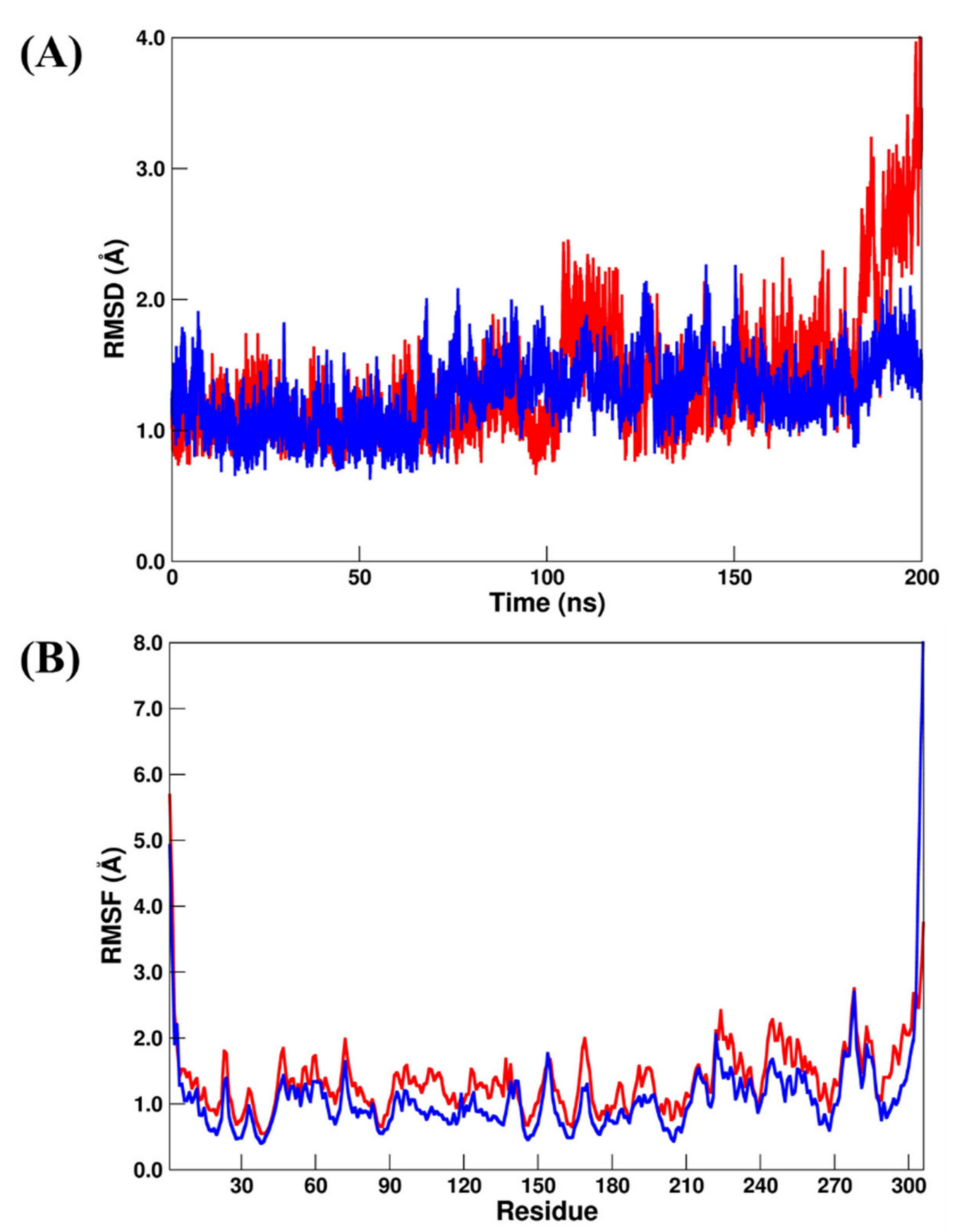
(**A**) RMSD and (**B**) RMSF plots for Mpro–X77 (red line) and Mpro–OUB (blue line). All values are reported in Å.

Here, Mpro–X77 (crystal reference) and Mpro–OUB (repurposed system) were submitted to 200 ns production MD simulations. For the RMSD analysis (Figure 4A), the values for Mpro–X77 and Mpro–OUB changed from 1.37 ± 0.48 Å to 1.27 ± 0.25 Å, respectively. A similar trend was found for the RMSF analysis (Figure 4B), where the values changed from 1.37 ± 0.54 Å to 1.10 ± 0.71 Å. Both results suggest excellent structural stabilization during all the MD production scales. Particularly, Mpro–X77 system showed an increase of about 1 Å on the last 20 ns MD simulation. However, it did not affect the binding of the crystal inhibitor (X77). Our findings are in good concordance with a previous computational study involving a drug repurposing analysis for the Mpro system [35]. Consequently, OUB can be considered as a dual inhibitor of SARS-CoV-2 through in silico screening against two SARS-CoV-2 targets: Mpro and PLpro. 

#### 2.3.3. RdRp Systems 

**Figure 5:**
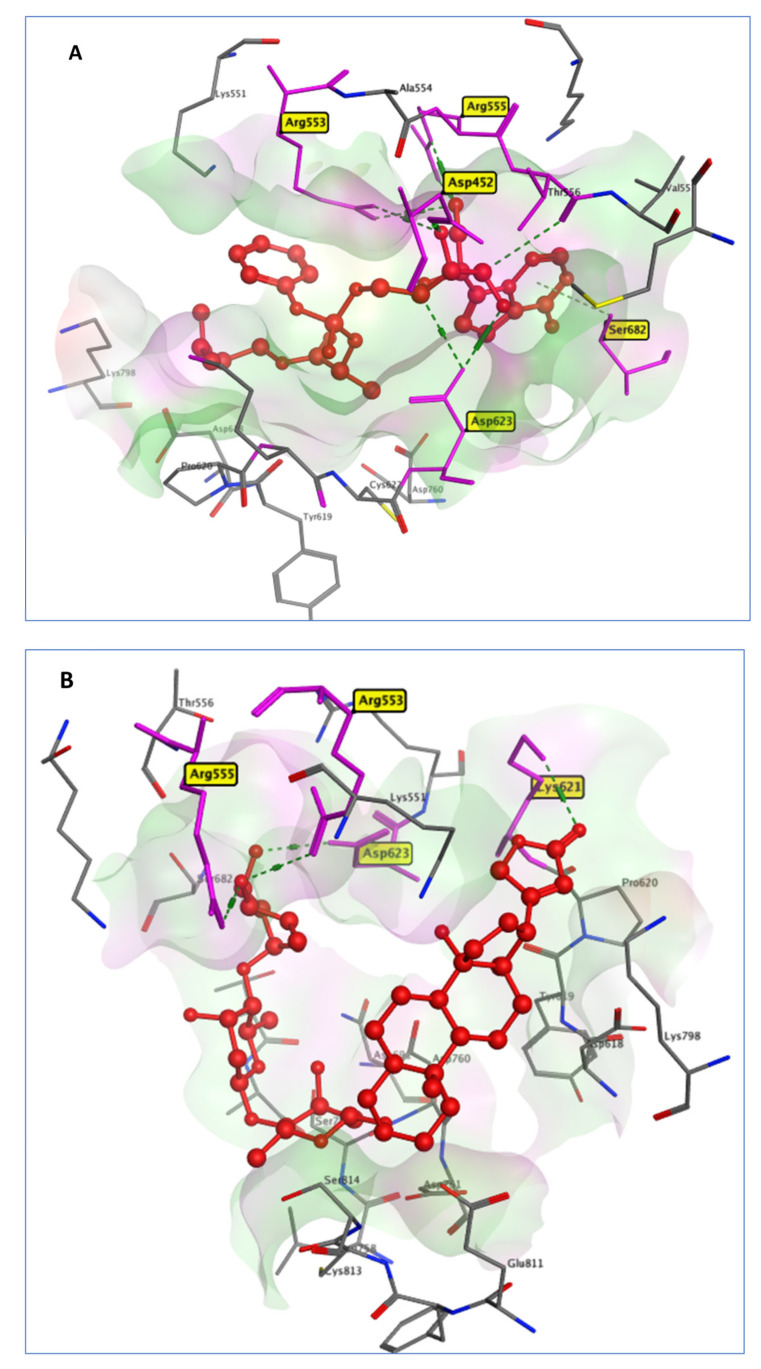
3D interactions between RDV (**A**) and DIG (**B**) with 7BV2 active site residues.

Natural cardiac glycosides, such as Digoxin, Digitoxin, and Proscillaridin, can be utilized to treat congestive heart failure and cardiac arrhythmia. The virtual screening of our selected drugs indicated the affinity of these drugs for SARS-CoV-2 RNA-dependent RNA polymerase (RdRp) (PDB code: 7BV2). The RdRp enzyme allows the viral genome to be transcribed into new RNA copies using the host cell’s machinery [38]. RdRp is required for the RNA virus life cycle but has no homolog in the host cell. This allows for the development of antiviral drugs and minimizes the risk of a protein in human cells being impacted [39]. Remdesivir, a repositioned drug, was taken as a standard. The in silico docking of RDV on (RdRp) protein hydrogen bonded to the active site residues Asp730, Arg588, and Arg523 with a docking score of −7.8 Kcal/mol. DIG had a binding pattern to RdRp similar to RDV as it was connected to the active site residues Arg553, Arg555, Lys621, and Asp623 through hydrogen bonds with an almost similar binding score of −7.69 Kcal/mol, compared to the RDV docking score of −7.8 Kcal/mol, as illustrated in Figure 5.

**Figure 6:**
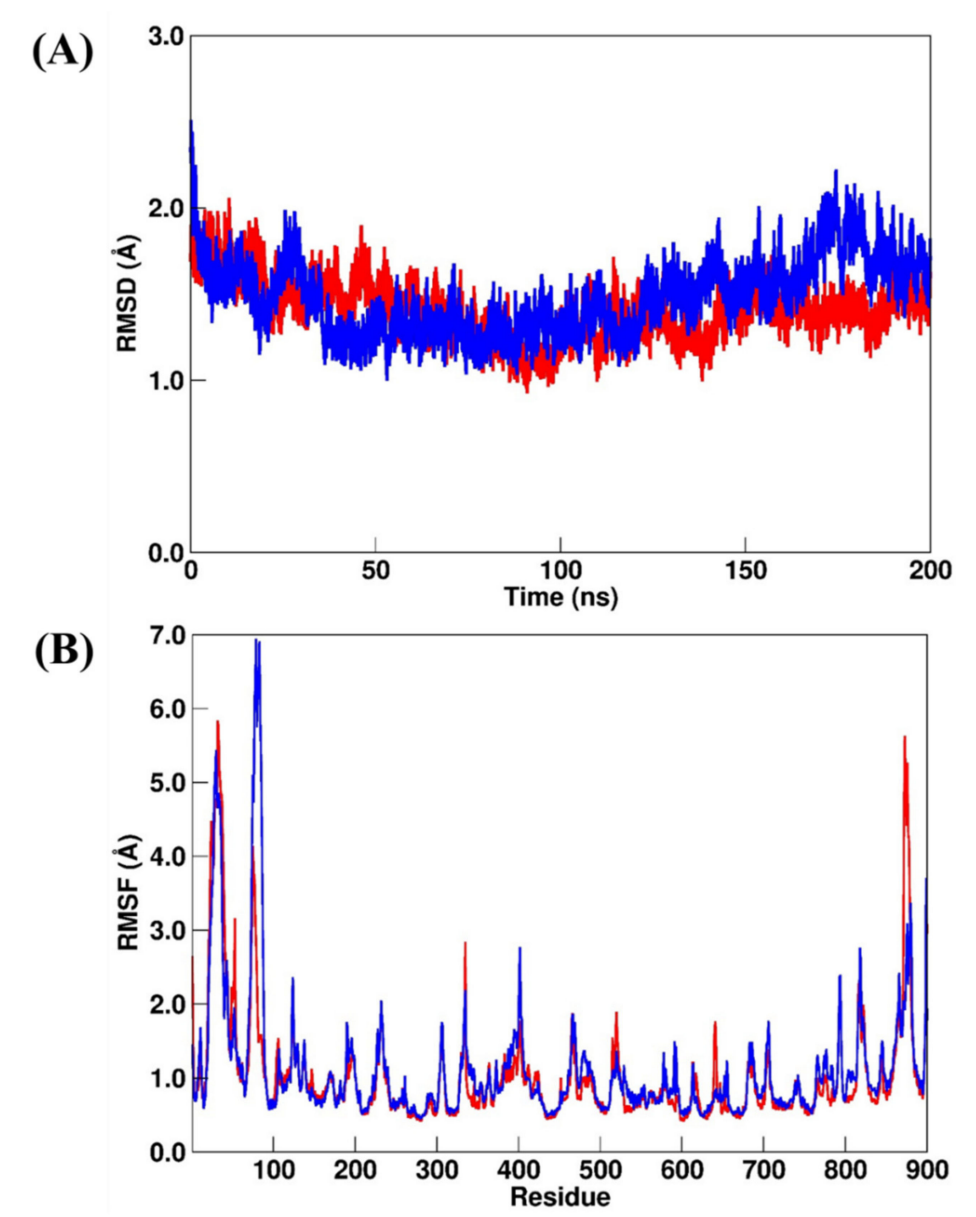
(**A**) RMSD and (**B**) RMSF plots for RdRp–RDV (red line) and RdRp–DIG (blue line). All values are reported in Å.

Recently, MD simulation studies for RdRp have been carried out using RDV as a bound inhibitor [40,41]. Particularly, Srivastava et al. [40] used different RDV models (named monophosphate and triphosphate). On the other hand, Koulgi et al. [41] used RDV before any metabolic change during 100 ns of MD simulations. Here, we chose to follow Koulgi’s proposal, but we performed 200 ns of MD simulations. According to our RMSD and RMSF analysis, the RdRp–RDV (crystal reference) and RdRp–DIG (repurposed) systems had similar structural stabilization. The RMSD values (Figure 6A) for RdRp–RDV and RdRp–DIG changed from 1.40 ± 0.17 Å to 1.48 ± 0.22 Å, respectively, while the RMSF values (Figure 6B) changed from 1.08 ± 0.75 Å to 1.23 ± 0.80 Å, respectively.

#### 2.3.4. AAK1 Systems 

**Figure 7:**
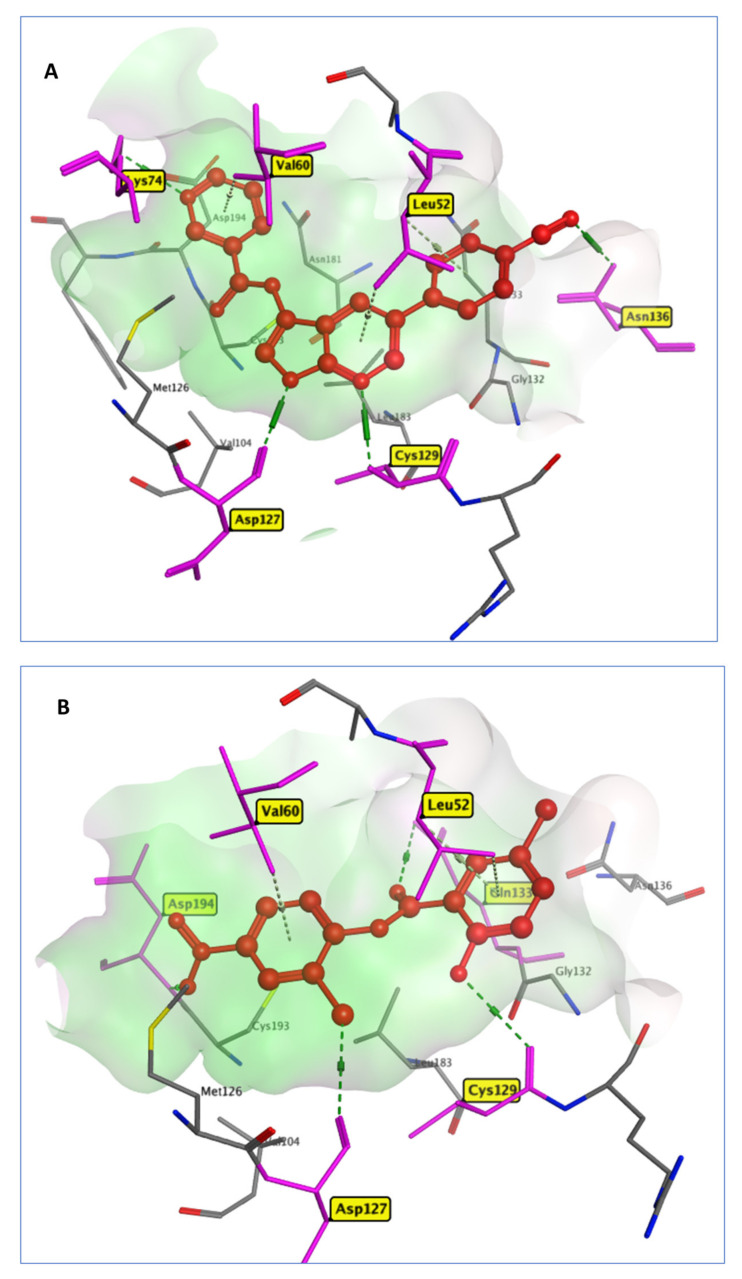
3D model of the interactions between crystallized ligand LKB (**A**) and NIS (**B**) in 5L4Q active site residues.

Niclosamide (NIS) is an antiparasitic drug that prevents the tapeworm from absorbing glucose, as well as preventing oxidative phosphorylation and anaerobic metabolism [42]. In comparison to the cocrystallized inhibitor (AE = −11.72 Kcal/mol), it was able to robustly bind to the AAK1 active site (AE = −11.84 Kcal/mol). AKK1 is a key kinase of receptor-mediated endocytosis found in the host cell, which regulates viral entrance and processing. Previous research has found that inhibiting AKK1 selectively can be helpful in SARS-CoV-2 treatment, either by blocking viral endocytosis or lowering the presence of pro-inflammatory molecules (IFN-γ and IL-6) [43]. Due to its affinity for the AP2-associated protein AAK1, Baricitinib, an efficient AAK1 and GAK inhibitor, has recently been discovered to have antiviral effects, reducing SARS-CoV-2 endocytosis [44,45]. From the docking results, NIS interacted with the AKK1 active site through four hydrogen bonds between carbonyl, chloride, phenolic hydroxyl, and nitro moieties and the active site residues Asp133, Lys127, Cys129, and Asp194, respectively. Further, it formed three π interactions with the amino acids Leu52 and Val60 (Figure 7), while the native ligand LKB formed four hydrogen bonds with Lys74, Asp127, Cys129 and Asn136 in addition to a Pi–H interaction with the amino acids Leu52 and Val60.

**Figure 8:**
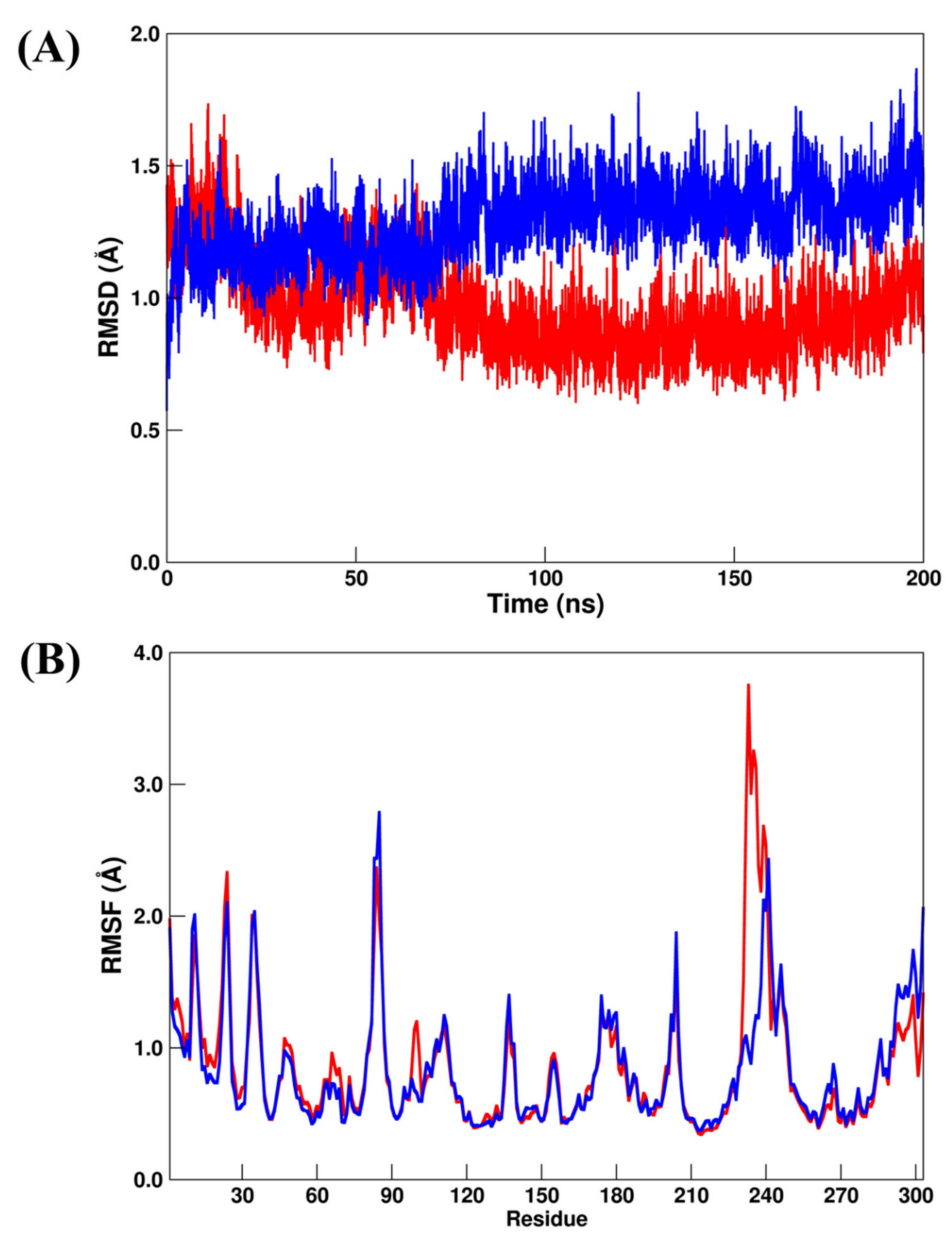
(**A**) RMSD and (**B**) RMSF plots for AAK1–LKB (red line) and AAK1–NIS (blue line). All values are reported in Å.

The structural analysis for the AAK1 systems by considering the RMSD and RMSF calculations for AAK1–LKB (crystal reference) and AAK1–NIS (repurposed) systems suggested that they were more structurally stable than the previous simulated systems. The RMSD values (Figure 8A) for AAK1–LKB and AAK1–NIS changed from 0.96 ± 0.17 Å to 1.29 ± 0.51 Å, respectively, while the RMSF values (Figure 8B) changed from 0.86 ± 0.51 Å to 0.83 ± 0.43 Å, respectively. However, by comparing the RMSF plots of AAK1–LKB and AAK1–NIS, we can observe a suitable divergence in the region that comprises residues from Trp214 to Tyr260. For the previous simulated systems, no similar differences were found.

### 2.4. Binding Free Energy Calculations 

As described in the Material and Methods section, relative binding free energies for all simulated systems were computed using the MM/GBSA approach [46,47,48] as implemented in *MMPBSA.py* [49] and are presented in Table 3. A single MD trajectory of the bound complexes was used to calculate the relative binding free energy ($\Delta G_{bind}$); the $\Delta E_{int}$ term (in Equation (3)) was canceled once the changing energy between complex systems and their components were computed using the same MD ensemble [47,48].

All the binding free energy terms are also listed in Table 3. For the PLpro systems, we can see that in PLpro–GRM, the vdW term ($\Delta E_{vdW}$) was the most important component for binding free energy followed by the polar term ($\Delta G_{polar}$). The PLpro–SLM showed similar values of $\Delta E_{vdW}$ and $\Delta G_{bind}$ when compared with PLpro–GRM. However, a significant difference can be observed in electrostatic ($\Delta E_{elec}$) and polar terms, where the first term increased from 0.70 Kcal/mol to 52.9 Kcal/mol for PLpro–GRM and PLpro–SLM, respectively, while the second term ($\Delta G_{polar}$) decreased from 13.9 Kcal/mol to −41.4 Kcal/mol, respectively. The most interesting result can be observed for PLpro–OUB, where the $\Delta E_{elec}$ and $\Delta G_{polar}$ appeared to be the most important terms for OUB binding with the catalytic site of the PLpro enzyme. As can be seen in Table 3, there was a decrease in the $\Delta E_{elec}$ value from 0.70 Kcal/mol to −41.4 Kcal/mol for PLpro–GRM and PLpro–OUB, respectively. In addition, the $\Delta G_{polar}$ term increased from 13.9 Kcal/mol to 61.9 Kcal/mol. These changes resulted in a large decrease in $\Delta G_{bind}$ from −29.7 Kcal/mol to −37.4 Kcal/mol, respectively. Our findings suggest that OUB is a more favorable inhibitor than the crystal inhibitor (GRM). This agrees with its reported activity against SARS-CoV-2, as well as the IC_50_ values in Vero and Calu cells (Table 1).

In the Mpro systems, Mpro–X77 showed the lowest $\Delta G_{bind}$ value (−42.8 Kcal/mol) in comparison with the Mpro–OUB (−28.7 Kcal/mol) systems. Particularly, the increase in the $\Delta E_{vdW}$ and $\Delta G_{polar}$ terms from −52.9 Kcal/mol to −41.9 Kcal/mol and from 39.1 Kcal/mol to 47.7 Kcal/mol, respectively, for Mpro–X77 and Mpro–OUB, explain the large increase in $\Delta G_{bind}$ for the Mpro systems.

Interestingly, for the RdRp systems, the RDV and DIG inhibitors showed similar $\Delta G_{bind}$ values. However, the free energy components were very different. As can be observed in Table 3, the $\Delta E_{elec}$ and $\Delta G_{polar}$ showed significant changes between these compounds. The $\Delta E_{elec}$ increased from −219.8 Kcal/mol to −34.6 Kcal/mol for RDV and DIG, respectively. This occurred due to the highest electronic density of RDV, mainly due to the presence of its phosphate group. On the other hand, the $\Delta G_{polar}$ decreased from 250.2 Kcal/mol to 76.8 Kcal/mol for RDV and DIG, respectively. Probably, it resulted due to compensation for the electrostatic effects of the electronic density of the RDV compound. Besides, the $\Delta G_{bind}$ of RDV (−13.0 Kcal/mol) is close to the value found by Srivastava et al. [40] (−16.6 Kcal/mol), which means that our RDV model is similar to the monophosphate RDV model that was simulated previously.

For the AAK1 systems, AAK1–LKB and AAK1–NIS showed very similar $\Delta G_{bind}$ values (−41.4 and 39.2 Kcal/mol, respectively). These results suggest that NIS can bind the catalytic site of the AAK1 enzyme in a similar mode to the crystallized inhibitor (LKB). The main energetic differences were found in the $\Delta E_{elec}$ and $\Delta G_{polar}$ terms. The first term increased from −31.3 Kcal/mol to −22.0 Kcal/mol and the second decreased from 40.3 Kcal/mol to 29.1 Kcal/mol for AAK1–LKB and AAK1–NIS, respectively.

In summary, if we consider the contributions of different $\Delta G_{bind}$ components of crystal and repurposed systems, the $\Delta E_{vdW}$ is a crucial term of the difference in the binding affinity in most systems, which agrees with the previous repurposed study [35]. Interestingly, our findings for the most promising repurposed compound (OUB) suggest that it can bind the PLpro enzyme with a binding affinity driven by the electrostatic interactions, with more evidence than any other repurposed system.

**Figure 9:**
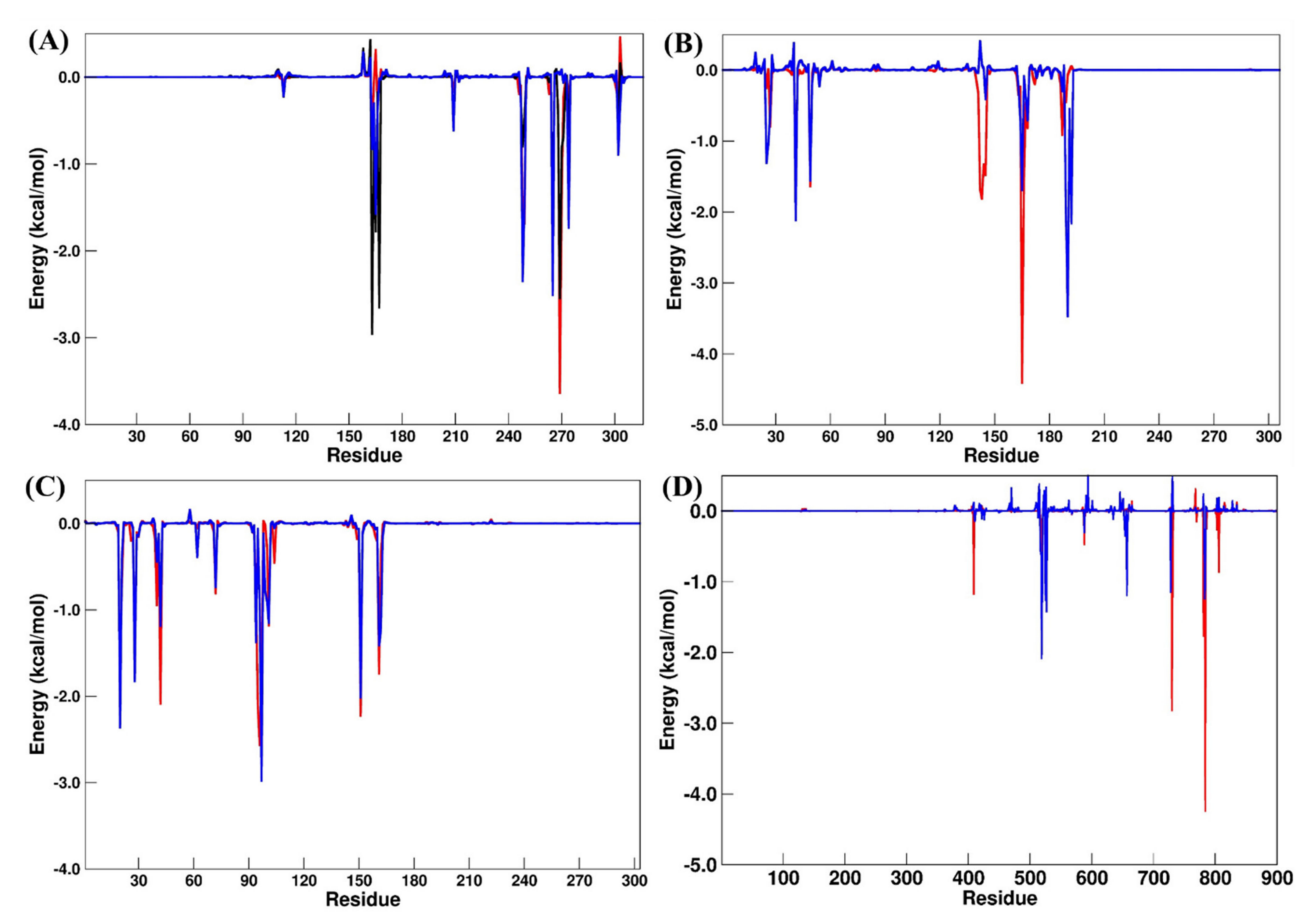
Residual decomposition plot for (**A**) PLpro (GRM, red; OUB, blue; SLM, black), (**B**) Mpro (X77, red; OUB, blue), (**C**) AAK1 (LKB, red; NIS, blue) and (**D**) RdRp (RDV, red; DIG, blue) systems. All values are reported in Kcal/mol.

A residual decomposition analysis of $\Delta G_{bind}$ (Figure 9) was carried out to improve the understanding of the features that contributed to the recognition and binding in all the simulated systems. Here, any residue that contributed to the binding free energy values below −1.20 Kcal/mol was included as an important residue in the binding process.

For the PLpro systems (Table 4), the main residual differences occurred for Leu163, Asp165, and Arg167 when PLpro–GRM and PLpro–OUB were compared, while Asp165, Tyr269, and Gln270 had suitable differences in the PLpro–SLM system. These results suggest that the electrostatic shift caused by Asp165 and Arg167 explains the favorable binding energy found for the PLpro–OUB system.

In the Mpro systems, the increasing values for Asn142, Gly143, Ser144, Cys145, Met165, and Glu166 appear to explain the binding differences found for Mpro–X77 and Mpro–OUB, although an energetic decrease was observed for Thr190 and Gln192. On the RdRp systems, the main binding difference occurred in the electrostatic term; by considering the residual decomposition analysis, some polar residues had significant changes between the RDV and DIG systems. We can also highlight the increase in the residual energies in Glu730, Cys731, Glu812, and Ser815 from the RdRp–RDV to the RdRp–DIG system.

Finally, for the AKK1 systems, only Asp95 and Phe96 showed some significant energetic shifting, which was not enough to promote a large difference in the binding of NIS. These results suggest that both compounds (LKB and NIS) bind to the catalytic site of AKK1 with a similar binding affinity trend.

## 3. Computational Methods

### 3.1. FDA-Approved Small Molecule Selection Rationale

The PubChem database (https://pubchem.ncbi.nlm.nih.gov/ (accessed on 4 January 2022)) [50] (was used to retrieve the FDA-approved drugs database. Molecular Operating Environment (MOE) platform was utilized to perform energy optimization for 3D structures of each compound using the MMFF94x forcefield (with the gradient set to a root mean square (RMS) of 0.05 kcal/mo1). The structures of the drugs are shown in Scheme 1.

### 3.2. Target Selection from Identified SARS-CoV-2 Protein Crystal Structures

Although any NSP may be included as a therapeutic target, the crystal structure and reported ligand, as well as its critical role in viral infection, greatly boost the success chances. Based on this hypothesis and focusing on SARS-CoV-2 reported studies, it is worth noting the NSP structures accessible for this virus: the main protease (Mpro, NSP5) [51,52], the papain-like protease (PLpro, NSP3) [10,53], the RNA-dependent RNA polymerase (RdRp, NSP12) [54] in complex with cofactors NSP7 and NSP8, as well as a human target, adaptor-associated kinase 1 (AAK1) [14,55], which was involved in a molecular docking study.

### 3.3. In Silico Docking Protocol Validation

Before docking, native ligands in the active region of the active domains were re-docked to verify the docking process, which resulted in a docking pose with certain RMSD values. For the SARS-CoV-2 Mpro, PLpro, RdRp, and AAK1, and their respective cocrystallized ligands, the computed RMSD values were 1.483, 0.665, 0.883, and 0.321 Å, respectively. The validated docking methodologies were then utilized to investigate the ligand–protein interactions of the tested drugs at the target active site to anticipate their binding mode. The measured RMSD values of the tested drugs indicated the efficiency and validity of the docking process.

### 3.4. Molecular Docking

The molecular modeling simulations were performed using the Molecular Operating Environment (MOE, 2020.09) software. With the MMFF94x force field, all minimizations were carried out with MOE until an RMSD gradient of 0.05 Kcal/mol was reached, and the partial charges were calculated automatically. Three viral SARS-CoV-2 proteins: (i) The SARS-CoV-2 Mpro (PDB ID: 6W63, resolution: 2.16 Å), (ii) PLpro (PDB ID: 3MJ5, resolution: 2.65 Å) [56], (iii) RdRp (NSP 12, PDB ID: 7BV2, resolution: 2.50 Å) [57], in addition to a host protein (iv) AAK1 (PDB ID: 5L4Q, resolution: 1.97 Å) [58] were downloaded from the RCSB Protein Bank Database. The proteins were selected according to their therapeutic potential. First, water molecules were removed. Then, for the docking study, all protein preparation was performed in MOE with the default options.

The generated ligand conformations were docked using a validated docking methodology, and the Triangle Matcher placement method was used and scored based on London dG. The top best 50 poses were refined, and the energy was minimized in the binding site using the induced fit method and then reranked with the GBVI/WSA scoring function [59]. In all the selected systems, the cocrystallized ligands were used as a reference for the selected FDA drugs during the molecular docking procedures. Afterwards, the 3D and 2D ligand–receptor binding patterns of the selected compounds were investigated.

### 3.5. Molecular Dynamic (MD) Simulations and Binding Free Energy

As a powerful tool to avail in silico, the stability of the above-repurposed systems, classical molecular dynamics (MD) simulations were carried out by using the PMEMD module [60] of the Amber20 package. Preliminary, all ligands were structurally optimized at the quantum mechanics (QM) level by applying the Hartree–Fock (HF) method with 6-31G** as implemented into the Gaussian09 program [61]. Afterwards, MM charges for each molecule were computed using the restrained electrostatic potential (RESP) method [62] by using the antechamber module of AmberTools20 [63]. The GAFF [64] and ff19SB [65] MM parameter sets were chosen for ligands and enzymes, respectively. Particularly, for 3MJ5 and 7BV2 systems, the Zinc AMBER force field (ZAFF) [66] was selected to simulate their metal centers. In addition, the SWISS-MODEL web server [67] was used to add missing amino acid residues into the 7BV2 system. The protonation state of the ionizable residues of all systems, at pH = 7, was calculated on PROPKA 3.1 version [68] and protons were added by the tleap module of AmberTools20 [63]. Each system was solvated by the TIP3P water model [69], extending 8 Å away from the solute atoms. Next, the appropriate counter-ions were added in order to neutralize the charges in the solvated system.

Each solvated system was energetically minimized to avoid bad atomic contacts using the PMEMD module. The minimization was carried out in four successive stages by applying 5000 steepest descent steps followed by 5000 steps of the conjugate gradient method, where restraints were removed during the process. After successful minimization, each system was slowly heated up to 300 K over 100 ps under constant volume, where the solute was restricted with the positional restraints of 50 Kcal/mol·Å2. Next, maintaining the same solute restraints, 200 ps of MD was performed at the NPT ensemble (p = 1 atm and T = 298 K). Then, the force constant of the restraints was slowly removed over the eight stages of equilibration; each stage was carried out for 100 ps under the NPT ensemble. Finally, a 200 ns unrestrained MD simulation (named “production stage”) was performed for each equilibrated system. The SHAKE algorithm [70] with the time step of 2 fs was applied for all the hydrogen bonds. The non-bonded cut-off set to 10 Å was used for the non-polar and polar interactions calculated using the Particle Mesh Ewald (PME) method [71]. The same MD protocol was used for all systems.

The structural analysis of all the simulated systems was evaluated by computing the root mean square deviation (RMSD) and root mean square fluctuation (RMSF) of the backbone atoms (Cα, N, O, C). Particularly, the RMSD calculations for each ligand were computed by considering only their respective heavy atoms. The trajectories were aligned by the main-chain atoms of the average structures from production stages by using the CPPTRAJ module [72].

The CPPTRAJ module [72] was used to extract 10 ns (a total of 1000 representative snapshots) from the production stage of the MD simulations on each system to be selected for the binding free energy calculations using the MM/GBSA approach [46,47,48], which was implemented into the *MMPBSA.py* module [49] of AmberTools20. The main equations of the chosen approach can be summarized as follows:

(1) $$\Delta G_{bind}=\Delta G_{complex}-\left(\Delta G_{receptor}+\Delta G_{ligand}\right)$$

(2) $$\Delta G_{bind}=\Delta E_{MM}+\Delta G_{SOLV}-T\Delta S$$

(3) $$\Delta E_{MM}=\Delta E_{int}+\Delta E_{elec}+\Delta E_{vdw}$$

(4) $$\Delta G_{SOLV}=\Delta G_{GB}+\Delta G_{SA}$$

where $\Delta G_{complex}$, $\Delta G_{receptor}$ and $\Delta G_{ligand}$ indicate the free energies of the complex, the receptor, and the ligand, respectively (Equation (1)). The $\Delta G_{bind}$ is obtained from the gas-phase MM energy ($\Delta E_{MM}$), solvation energy ($\Delta G_{SOLV}$) and the entropic term (−TΔS) (Equation (2)). The $\Delta E_{MM}$ includes the changes in the internal (bond, angles, and dihedral energies) ($\Delta E_{int}$), electrostatic ($\Delta E_{elec}$) and van der Waals ($\Delta E_{vdw}$) contributions (Equation (3)). Here, as a single-trajectory scheme was used for the binding free energy calculations, the $\Delta E_{int}$ is equal to zero. The $\Delta G_{SOLV}$ is the sum of the polar ($\Delta G_{GB}$) and non-polar ($\Delta G_{SA}$) energies for $\Delta G_{bind}$ (Equation (4)). To reduce the computational cost, the entropic term (−TΔS) was not computed into the binding free energy calculations [47,48]. Furthermore, a per-residual decomposition analysis was included to avail the relative contribution of each amino acid residue [46]. This method has been frequently applied as a useful tool in SARS-CoV-2 drug design studies [35,40,41,73,74,75,76].

## 4. Conclusions and Prospects

A set of comprehensive docking approaches and molecular dynamics simulations were performed to find viable drugs for inhibiting SARS-CoV-2 targets. Accordingly, based on docking and the MD simulation results, Digitoxin was postulated as an effective RNA-dependent RNA polymerase inhibitor compared to Remdesivir as a known inhibitor. It was concluded that Salinomycin may act as a PLpro inhibitor, while Ouabain can share the activity with Salinomycin and Proscillaridin as a dual inhibitor of both PLpro and Mpro SARS-CoV-2, respectively. In addition, Niclosamide was proposed to be an inhibitor for adaptor-associated kinase 1. The insights provided by the present study may substantiate the valuable exploration and development of anti-SARS-CoV-2 therapeutic agents from FDA-approved drugs, to be used as leads for further drug development. Furthermore, drug repositioning can be considered an efficient tool as a source of new analogs to help defeat COVID-19.

## Figures and Tables

**Table 1:** FDA-approved drugs are considered tested ligands that were selected for molecular docking. Drug generic names, clinical use, and reported activity against SARS-CoV-2.

Drug Generic Name	Clinical Use	(IC_50_, μM) in Vero Cell	(IC_50_, μM) in Calu Cell
Ouabain (OUB)	Cardiovascular agents	<0.097	0.1
Digoxin (DGX)	Cardiovascular agents	0.19	0.72
Digitoxin (DIG)	Cardiovascular agents	0.23	0.16
Salinomycin (SLM)	Antibacterial agents	0.24	0.5
Niclosamide (NIS)	Antiparasitic agents	0.28	0.84
Proscillaridin (PRO)	Cardiovascular agents	2.04	5.95

**Table 2:** Affinity energy (AE) values (Kcal/mol) of binding for FDA-approved repositioned drugs to selected SARS-CoV-2 target proteins.

Drug Name	PLpro	Mpro	RdRp	AAK1
Ouabain (OUB)	−9.52	−8.21	−6.35	−8.17
Digoxin (DGX)	−7.88	−7.64	−7.63	−9.08
Digitoxin (DIG)	−8.01	−6.56	−7.69	−9.9
Salinomycin (SLM)	−9.02	−4.97	−5.19	−9.05
Niclosamide (NIS)	−6.43	−5.87	−5.66	−11.84
Proscillaridin (PRO)	−6.22	−7.28	−7.11	−8.25
Cocrystallized ligand GRM	−8.46			
Cocrystallized ligand X77		−8.05		
Cocrystallized ligand RDV			−7.8	
Cocrystallized ligand LKB				−11.72

**Table 3:** Binding free energy ($\Delta G_{bind}$) values and their components for the crystal and repurposed systems by MM/GBSA. The errors were computed by using the single-trajectory protocol [48]. All values are reported in Kcal/mol.

System	$\Delta E_{vdW}$	$\Delta E_{elec}$	$\Delta G_{polar}$	$\Delta G_{non-polar}$	$\Delta G_{bind}$
GRM–PLpro	−39.7 (0.1)	0.7 (0.1)	13.9 (0.1)	−4.6 (0.1)	−29.7 (0.1)
OUB–PLpro	−38.2 (0.2)	−55.4 (0.3)	61.9 (0.2)	−5.7 (0.1)	−37.4 (0.2)
PLpro–SLM	−36.7 (0.1)	52.9 (0.2)	−41.4 (0.2)	−4.6 (0.1)	−29.8 (0.1)
Mpro–X77	−52.9 (0.1)	−22.8 (0.2)	39.1 (0.1)	−6.2 (0.1)	−42.8 (0.1)
Mpro–OUB	−41.9 (0.1)	−29.3 (0.2)	47.7 (0.1)	−5.2 (0.1)	−28.7 (0.1)
RdRp–RDV	−36.9 (0.1)	−219.8 (0.8)	250.2 (0.7)	−6.5 (0.1)	−13.0 (0.1)
RdRp–DIG	−50.6 (0.1)	−34.6 (0.3)	76.8 (0.3)	−6.3 (0.1)	−14.7 (0.1)
AAK1–LKB	−44.7 (0.1)	−31.3 (0.1)	40.3 (0.1)	−5.7 (0.1)	−41.4 (0.1)
AAK1–NIS	−41.0 (0.1)	−22.0 (0.1)	29.1 (0.1)	−5.3 (0.1)	−39.2 (0.1)

**Table 4:** Residual decomposition analysis of binding free energies. All values are reported in Kcal/mol.

PLpro systems			
	**GRM**	**OUB**	**SLM**
Leu163	−0.68	−2.96	−0.83
Asp165	0.31	−1.78	−1.58
Arg167	−0.12	−2.65	−0.02
Pro248	−1.60	−0.80	−2.35
Tyr265	−2.15	−1.75	−2.51
Tyr269	−3.64	−2.55	0.03
Gln270	−1.47	−0.81	0.09
Mpro systems			
	**X77**	**OUB**	
His41	−1.55	−2.12	
Met49	−1.64	−1.56	
Asn142	−1.68	0.41	
Gly143	−1.81	0.07	
Ser144	−1.33	0.02	
Cys145	−1.48	−0.41	
Met165	−4.41	−1.69	
Glu166	−1.56	−0.09	
Gln189	−0.45	−1.98	
Thr190	−0.07	−3.47	
Gln192	0.05	−2.16	
RdRp systems			
	**RDV**	**DIG**	
Ser550	−0.25	−2.08	
Val558	0.00	−1.42	
Thr688	0.03	−1.20	
Glu730	−2.82	0.30	
Cys731	−1.45	0.50	
Glu812	−1.77	0.24	
Ser815	−4.24	−1.02	
AAK1 systems			
	**LKB**	**NIS**	
Leu20	−1.99	−2.36	
Val28	−1.65	−1.83	
Lys42	−2.09	−1.19	
Asp95	−2.11	−0.06	
Phe96	−2.56	−0.95	
Cys97	−1.69	−2.98	
Leu151	−2.23	−2.02	
Cys161	−1.74	−1.41	

## Data Availability

Any data related to this work might be available from the corresponding author.

## Sample Availability

Simulated PDB files are available from the authors.
